# Spectral band selection and ANIMR-GAN for high-performance multispectral coal gangue classification

**DOI:** 10.1038/s41598-024-58379-y

**Published:** 2024-04-02

**Authors:** Qingya Wang, Huaitian Hua, Liangliang Tao, Yage Liang, Xiaozheng Deng, Fen Yu

**Affiliations:** 1 College of Information Engineering, Jiujiang Vocational and Technical College, Jiujiang, 332000 Jiangxi People’s Republic of China; 2 School of Earth Science, East China University of Technology, Nanchang, 330013 Jiangxi People’s Republic of China; 3 Department of Mining Engineering, Shanxi Institute of Technology, Yangquan, 045000 Shanxi People’s Republic of China; 4 School of Automation Engineering, University of Electronic Science and Technology of China, Chengdu, 611731 Sichuan People’s Republic of China

**Keywords:** Multilevel residual network, CycleGAN, Peak signal to noise ratio, Structure similarity, Information technology, Engineering, Optical spectroscopy

## Abstract

Low-energy and efficient coal gangue sorting is crucial for environmental protection. Multispectral imaging (MSI) has emerged as a promising technology in this domain. This work addresses the challenge of low resolution and poor recognition performance in underground MSI equipment. We propose an attention-based multi-level residual network (ANIMR) within a super-resolution reconstruction model (ANIMR-GAN) inspired by CycleGAN. This model incorporates improvements to the discriminator and loss function. We trained the model on 600 coal and gangue MSI samples and validated it on an independent set of 120 samples. The ANIMR-GAN, combined with a random forest classifier, achieved a maximum accuracy of 97.78% and an average accuracy of 93.72%. Furthermore, the study identifies the 959.37 nm band as optimal for coal and gangue classification. Compared to existing super-resolution methods, ANIMR-GAN offers advantages, paving the way for intelligent and efficient coal gangue sorting, ultimately promoting advancements in sustainable mineral processing.

## Introduction

Coal remains a cornerstone of industry and a primary energy source, despite the environmental challenges posed by the production of coal gangue, a low-value byproduct necessitating efficient separation during beneficiation^1^. Traditional wet separation methods, while effective, contribute to significant water resource depletion and environmental pollution^2^. In contrast, dry coal separation methods prevalent in arid regions, like northwestern China, offer a less water-intensive alternative but often entail higher energy consumption and potentially reduced separation efficiency.

Non-destructive testing methods can be used to distinguish coal and gangue, and then remove gangue on the high-speed conveyor. Examples of these methods include dual-energy gamma-ray detection, X-ray detection^3^, spectral detection^4^, laser detection, and image detection. Radiation-based detection technology (gamma-ray and X-ray detection) yields good results, but the radiation risk severely limits its widespread application. Spectral detection yields good results, but the moisture on the surface has a significant impact on the detection results^4^. Based on image processing, coal gangue is not affected by moisture and has no radiation pollution. This has become one of the main research directions for coal gangue separation^5^.

Compared with the complexity of hyperspectral imaging, multispectral imaging equipment is relatively simple and reliable, the multispectral imaging (MSI) is based on fewer bands, which can achieve faster image acquisition speed and relatively lower costs. It has been successfully applied in various fields, such as agriculture^6^, food^7^, medicine^8^, and archaeology^9^. Research on the identification of coal and gangue using MSI has been extensively conducted. Yan et al.^10^ proposed an improved YOLOv5 structure for the spectral-based coal and gangue sorting, which selected five highly recognizable and correlated bands from 25 bands, thereby improving the recognition rate and detection speed. Hu et al*.*^11^ developed a new framework of Convolutional Neural Network (CNN) and used Bayesian optimization algorithm to optimize the parameters of CNN, which increased the accuracy of coal and gangue classification. These studies have developed some new classification algorithms. However, many research results have shown that MSI collected on conveyor belts is affected by factors such as high dust concentration, humidity, and temperature, which lead to low imaging resolution, distortion, and degradation, and thus affect the practical application effect of classification algorithms. Therefore, improving the image resolution is also one of the ways to solve the problem of coal and gangue identification technology^12^. However, there are currently few studies on super-resolution reconstruction of MSI for coal and gangue classification.

Image super-resolution reconstruction (SR) involves generating high-resolution images (HR) from multiple low-resolution images (LR)^13^. This technology finds significant applications in fields such as satellite remote sensing images, digital high definition, microscopy, video coding, and video surveillance. Deep learning is still the most commonly used method for image super resolution reconstruction. It is based on convolutional neural networks, residual networks, and sub-pixel convolution layers, and improves algorithm speed and accuracy by modifying network structures^14^, such as Super-resolution convolution neural network (SRCNN), Sparse coding based network (SCN), and Very deep networks for super-resolution (VDSR)^15^. However, these methods face challenges in terms of computational resource consumption, which may not align with engineering implementation needs. Additionally, the reconstructed images often exhibit excessive smoothness and lack texture information, which is crucial for coal and gangue identification^16^. Balancing image detail preservation and computational resource optimization is essential for cyclic network implementation, particularly when aiming to improve recognition accuracy and deploy the model in production environments with limited computing power. Generative Adversarial Networks (GANs) are a class of deep learning models that have gained significant attention in recent years due to their ability to generate realistic images, videos and other types of data^17^. Image super-resolution using GANs is a relatively new approach, but it has show promising results. Image super-resolution using GANs has a wide range of applications, including: medical imaging^18^, Security^19^, Entertainment. So GANs make them ideal for applications where high-quality images are essential.

In order to facilitate deployment on embedded devices, images are subjected to SR and then classified using mature and stable machine learning algorithms^20^. Random forest (RF) is a classical machine learning algorithm with advantages such as few parameter adjustments, ability to classify high-dimensional data, and parallel processing. There are many relevant research papers^21^ and some scholars have used RF for MSI recognition of coal and gangue, with experimental results showing that the RF achieves good classification effects^22^. Therefore, this paper plans to perform SR on actual coal and gangue MSI, and then use RF for classification, studying the modeling process and its effectiveness.

This paper contributes to coal gangue sorting by proposing a novel super-resolution reconstruction method based on an attention-enhanced, multi-level residual network (ANIMR-GAN). Our method significantly improves feature extraction and reconstruction, surpassing baseline methods in image quality and classification accuracy. We demonstrate the practical significance of our method using a real-world dataset of coal and gangue images.

## Materials and methods

### Multispectral data acquisition

**Figure 1:**
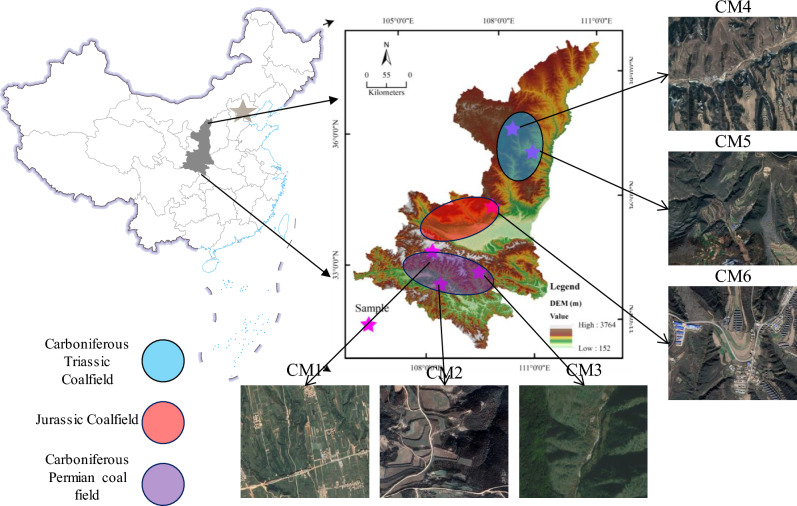
Select the location of the sample mining area, by selecting sampling areas from different geological regions and representing a wide range of coal and gangue types, including Carboniferous Triassic Coalfield, Jurassic Coalfield, Carboniferous Permian coal, CM1–CM6 is different coal mining. Data and reprinted from Refs.^24,25^, the DataV.GeoAtlas platform. Copyright (2022) (http://datav.aliyun.com/portal/school/atlas/area_generator, last access: 19 July 2022).

Shaanxi is an important coal-producing province in China, with areas such as Yan’an, Jingbian, and Yulin being the main coal mining regions^23^. 300 coal samples and 300 gangue samples were selected from the CM1–CM5 mining areas, as shown in Fig. 1. After collecting MSI, 300 images of coal samples and 300 images of gangue samples were obtained. These images were used for modeling and analyzing the performance of algorithms. In addition, 60 coal samples and 60 gangue samples were collected separately from the CM6 mining area, and MSI of the coal and gangue were obtained. These images were not used for modeling, but only for the validation of the final model. The size of the selected coal and gangue is 50–300 mm, mainly non-stick coal and long flame coal.

**Figure 2:**
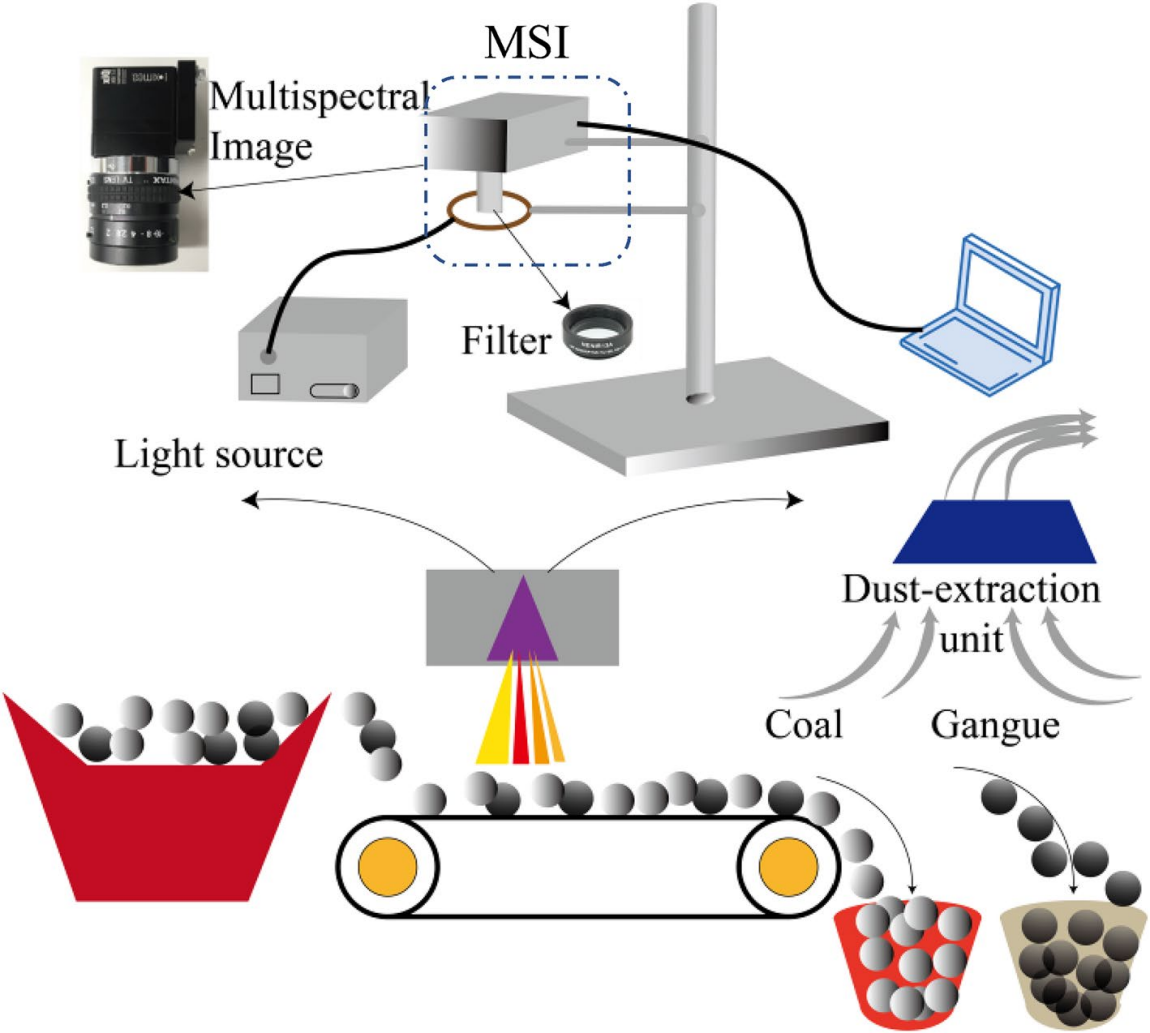
MSI acquisition system, the acquisition system includes the host, PC, light source, filter and some necessary accessories. The acquisition system is arranged on the conveyor belt of coal and gangue. There is a dust removal system arranged on the belt. The dust removal system realizes the dustless work equipment by suction pipe, centrifugal machine and filter, the separation of coal and gangue is realized by the function of automatic air compressor.

The MSI system for coal and gangue recognition is illustrated in Fig. 2, which consists of a light source and a spectrometer. The light source device is LS-LHA produced by SUMITA OPTICAL GLASS, Inc., with a maximum power of 150W and the corresponding average illumination is 406 k lx. The filtering device consists of two filters manufactured by Edmund Optics (United States), which allows only light with a wavelength in the range of [675 nm 975 nm] to pass. The multispectral imager is an MQ022HG-IM-SM5X5-NIR produced by XIMEA, Germany. It can obtain 25 spectral images in the wavelength range of [675 nm 975 nm], with a resolution of 409 × 216 Pixel for each image. For more detailed information, please refer to the References^26,27^.

**Figure 3:**
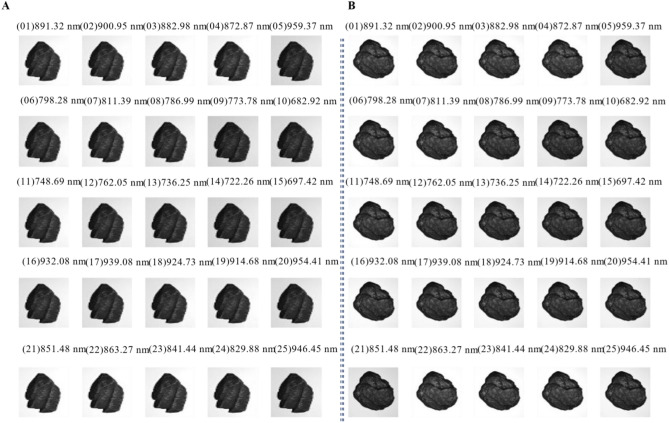
Multispectral data for coal and gangue, the spectral images of coal and gangue are displayed, where (01)–(25) represent the different wavelength bands of the spectral images in HSImager software. (**A**) Coal, (**B**) gangue.

The focal length and exposure time of the spectrometer were set to 3.2 mm and 85.03 ms, respectively. A total of 600 coal and gangue spectral imaging data were used for modeling, including 300 coal samples and 300 gangue samples. Due to the limited sample size, data augmentation techniques such as flipping and adding Gaussian noise were applied to the spectral images, resulting in a total of 3000 multispectral data. Among them, there were 1500 coal images and 1500 gangue images, with each multispectral data containing 25 spectral images. Figure 3 displays the spectral images of coal and gangue, where (01)–(25) represent the different wavelength bands of the spectral images in HSImager software.

**Figure 4:**
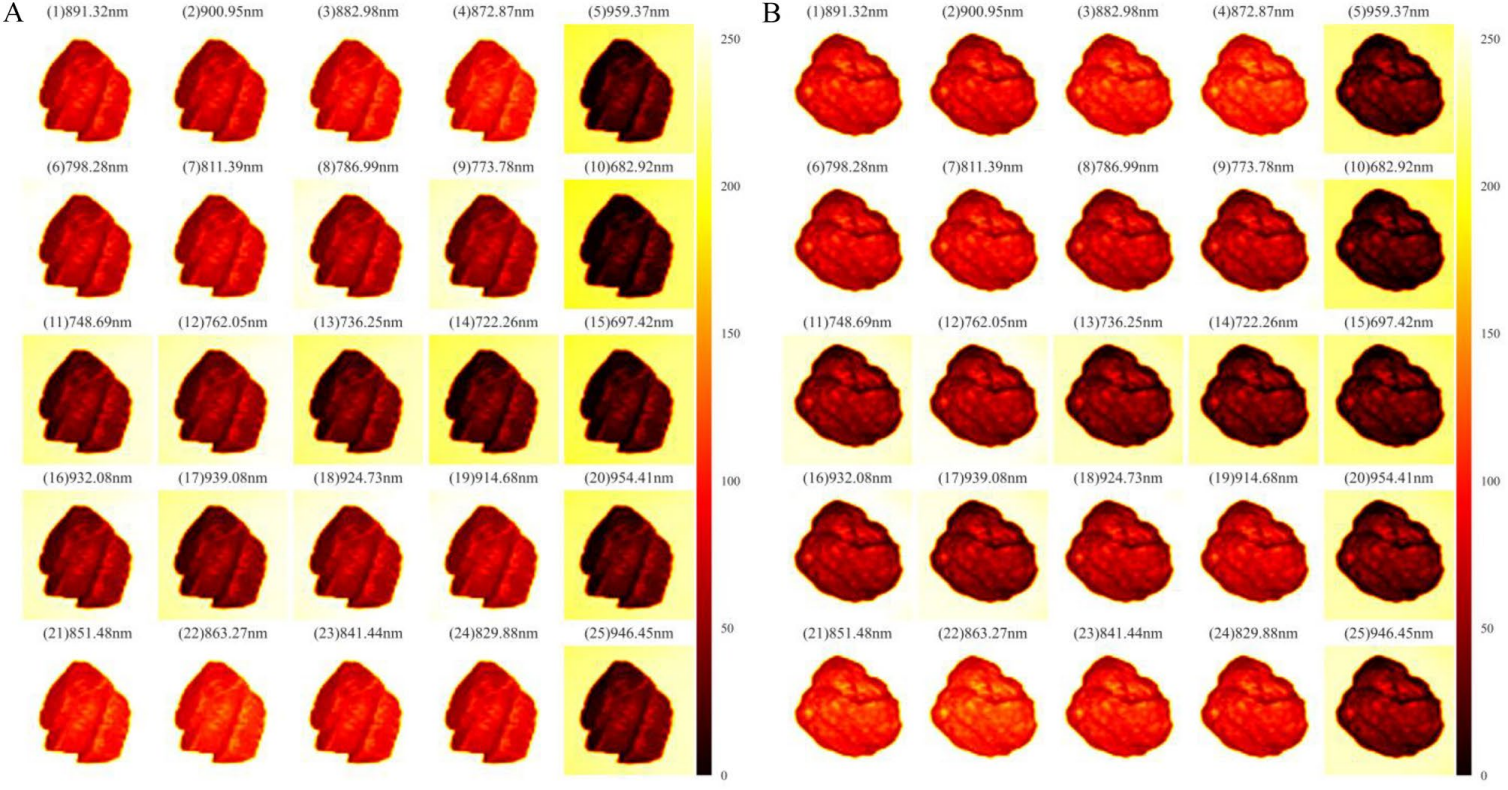
Spectral data of coal and gangue after treatment, the spectral images of coal and gangue are displayed, where (01)–(25) represent the different wavelength bands of the spectral images. (**A**) Coal, (**B**) Gangue.

The grayscale images are converted to three-channel thermodynamic diagrams, ranging from red to white for enhanced visualization. To standardize analysis, the images are resized to 400 × 400 pixels using quadratic interpolation. Figure 4 showcases the results—although visual differences between coal and gangue are more pronounced after processing, direct visual classification remains challenging. This highlights the need for super-resolution (SR) techniques combined with a suitable classification algorithm to optimize the mineral sorting process.

### Method

**Figure 5:**
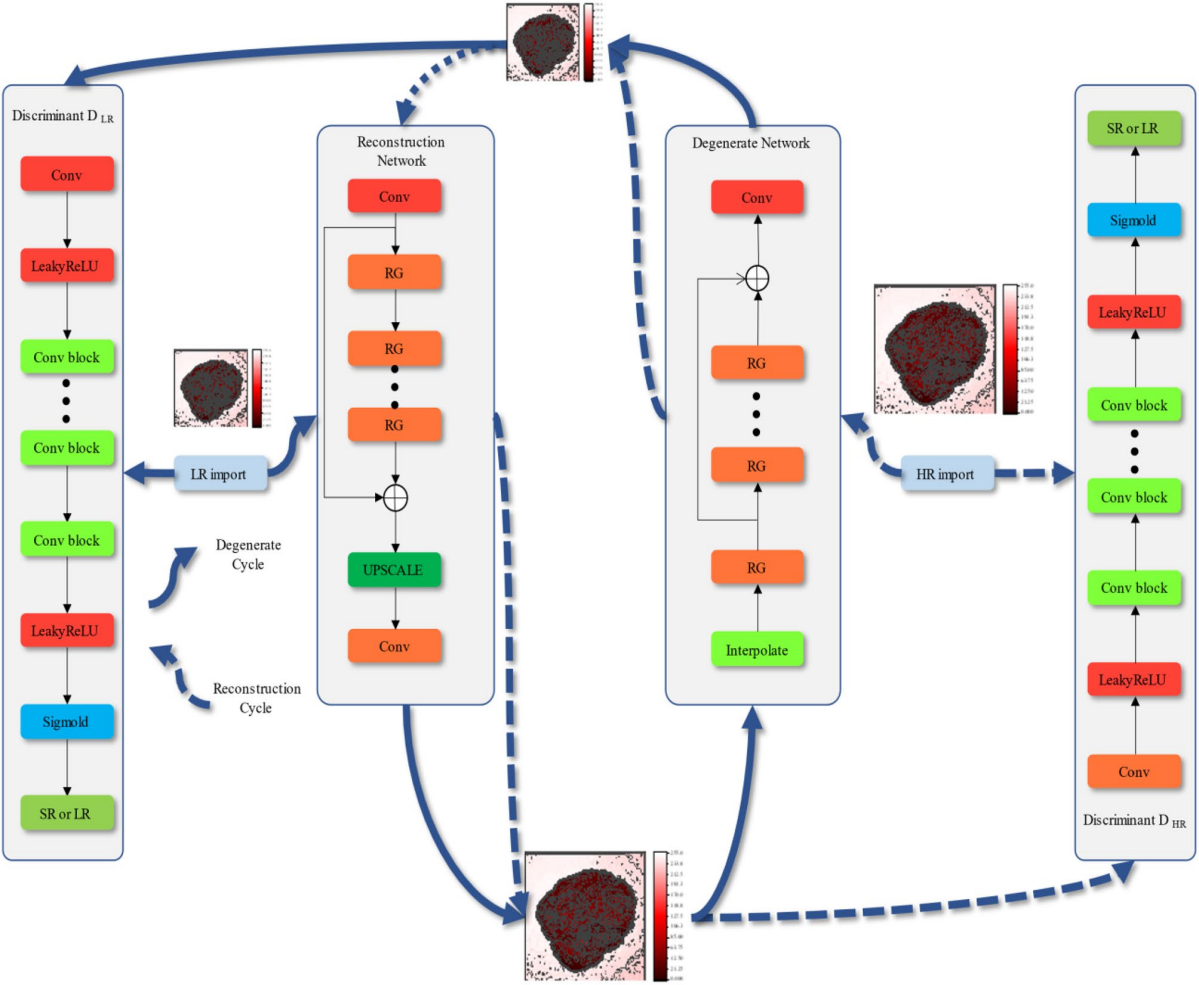
The overall ANIMR-GAN system architecture, *RG* residual group, The large heat maps represent the high-resolution images, the small heat maps represent the low-resolution images, a solid line represents a generative network, and a dashed line represents a reconstructed network, including a generator *G*, a degenerate network *F*, a low-resolution discriminator DL_R_, and a high-resolution discriminator DH_R_.

This paper proposes a SR method that integrates attention network and improved multi-level residual (ANIMR). This method introduces attention mechanism and utilizes the improved residual network to enhance the feature extraction ability. It has the dual advantages of low response time and high reconstruction image quality. The model consists of a generator and a discriminator, where the generator includes a reconstruction network *G* and a degradation network *F*, while the discriminator includes *DL*_R_ and *DH*_R_. *G* is responsible for reconstructing LR to HR, *F* is responsible for downsampling HR image into LR image, *D*_LR_ is responsible for distinguishing real LR images from degraded images, and *D*_HR_ is responsible for distinguishing real HR images from HR images obtained through reconstruction. The overall ANIMR-GAN system architecture is shown in Fig. 5.

#### Reconstruction network

**Figure 6:**
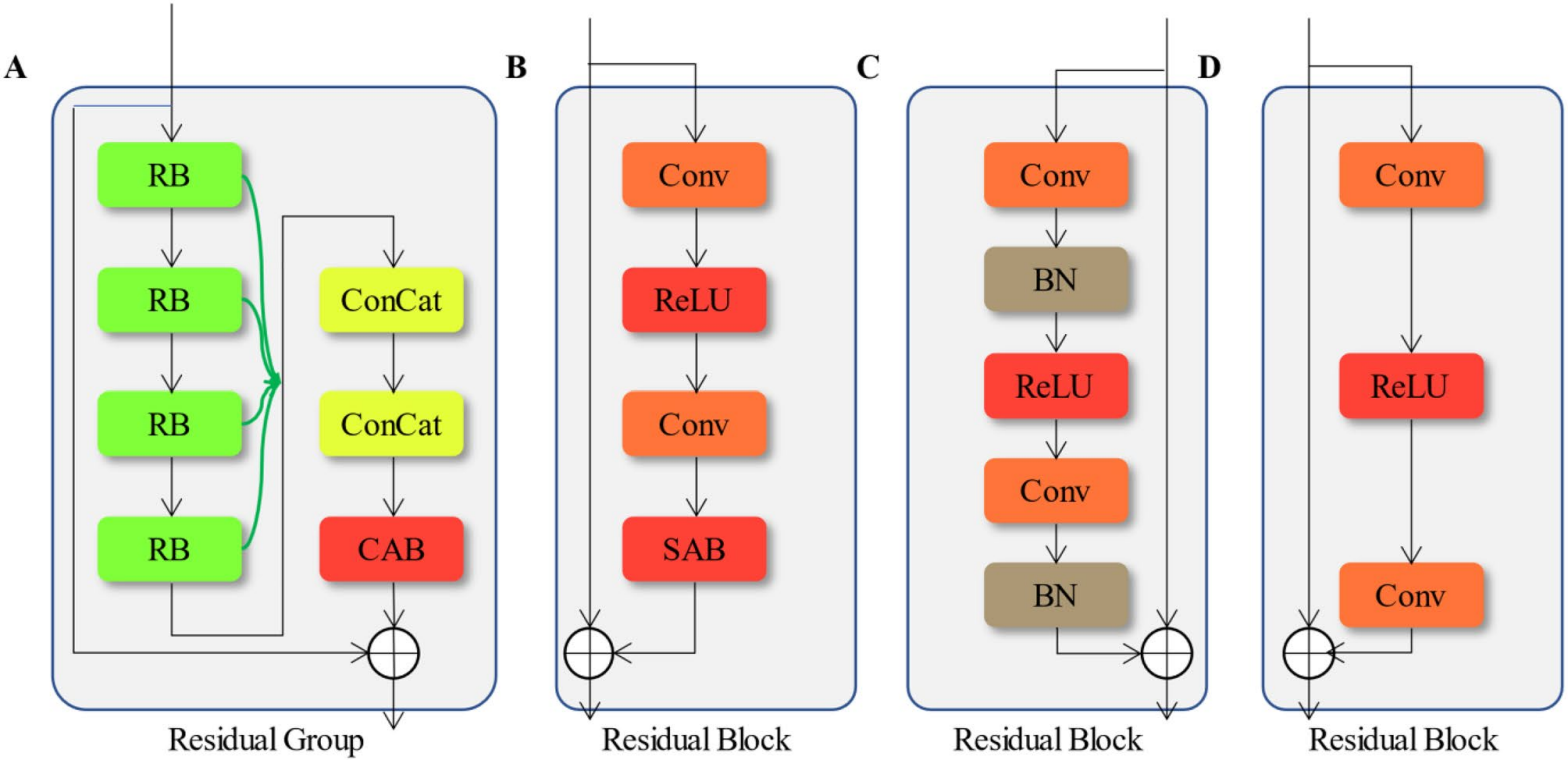
Structure of RG and RB. (**A**) ANIMR RG structure, (**B**) ANIMR RB structure, (**C**) SRResnet RB structure, (**D**) EDSR RB structure.

The task of *G* is to reconstruct the LR images into the HR images, which includes a low-level feature extraction module, a residual set, and an upsampling reconstruction module. The low-level feature extraction module extracts extremely low-level features from the three-channel image as the input for the subsequent network. The up-sampling and reconstruction module uses sub-pixel convolutional layers to reconstruct the LR images into the HR images, and uses one convolutional layer to restore the image to a three-channel image. The residual set consists of several residual groups (RG), which are used to learn the non-linear mapping relationship between LR and HR images. Each RG in the residual set has a structure shown in Fig. 6A, which consists of 4 residual blocks (RB), a channel attention block (CAB), and 2 convolution layers (ConCat) used to adjust channel numbers. Since low-level networks typically have more bottom-level information, to fully utilize this information, this paper proposes the concept of residual polymer(RP), which concatenates the output channels of the four RB, passes them through the channel attention block, and outputs them. This approach resolves the issue of the huge number of parameters caused by feature extraction in existing networks. For each input of the residual set, the output can be represented as shown in Eq. (1).

1 $$\tilde{X}_{{{\text{RG}}}} = X_{{{\text{RG}}}} + I_{{{\text{RG}}}} I_{{{\text{CAB}}}} \left[ {I_{{\text{c}}} \left( {K_{{{\text{RG}}1}} ,K_{{{\text{RG}}2}} ,K_{{{\text{RG}}3}} ,K_{{{\text{RG}}4}} } \right)} \right],$$

where *K*_RG1_, *K*_RG2_, *K*_RG3_, and *K*_RG4_ are the outputs of the four RB, *I*_c_ represents the concatenation of the outputs of the four RB, *I*_CAB_ represents the channel attention module, and *I*_RG_ is a 1 × 1 convolutional layer used to adjust the number of output channels.

In ANIMR, RB architecture is augmented with a spatial attention block (SAB) placed at the end of the block, while removing the BN layer, as illustrated in Fig. 6B. This study builds on the RB structure designed in SRResnet and VDSR (as shown in Fig. 6C,D)^28^ and introduces both channel attention module and SAB to enable the residual network to learn channel and spatial weights respectively, thereby enhancing the feature extraction capability of the network in different channel and spatial regions. The channel attention mechanism assigns higher weights to important channels, thus enhancing the feature extraction capability of the network without increasing the network width. The spatial attention mechanism assigns more attention to important high-frequency information such as texture and edges when allocating internal attention resources in the feature map. The addition of both channel attention and spatial attention modules results in reconstruction results that are more similar to the real image.

#### Degenerate network

**Figure 7:**
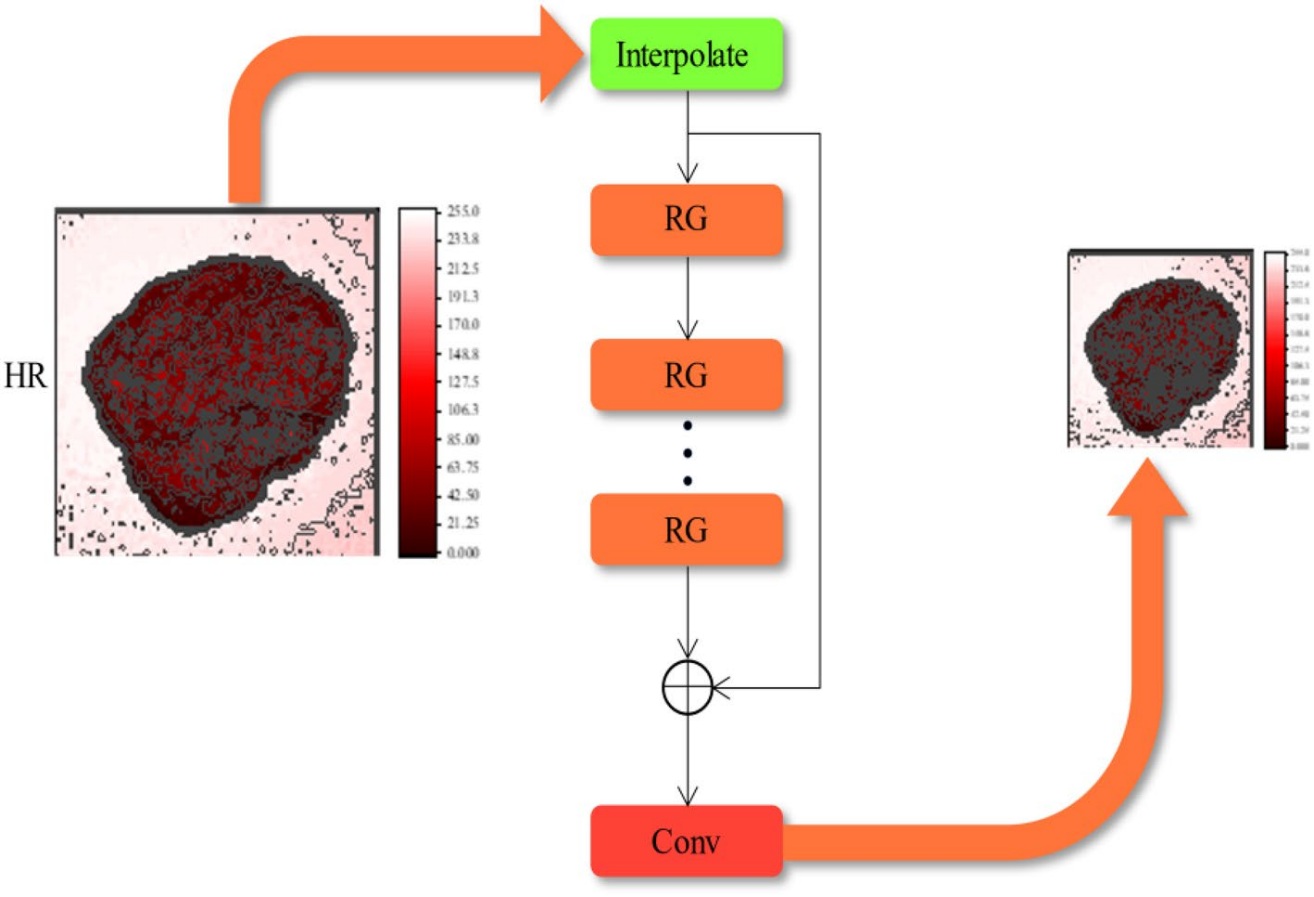
Degenerate network structure, HR is high resolution images, RG is Residual Group, the degenerate network first downsamples the HR image to the low-resolution space, then uses the residual set extracted in ANIMR to learn the mapping relationship between HR and LR.

The task of the degenerate network is to degrade the HR image to the LR image, which is the reverse process of the reconstruction network. As shown in Fig. 7, where Interpolate is an interpolation downsampling operation, the degenerate network first downsamples the HR image to the low-resolution space, then uses the residual set extracted in ANIMR to learn the mapping relationship between HR and LR, and finally reconstructs the predicted LR image through a convolutional layer.

#### DL_R_ and DH_R_ discriminator

**Figure 8:**
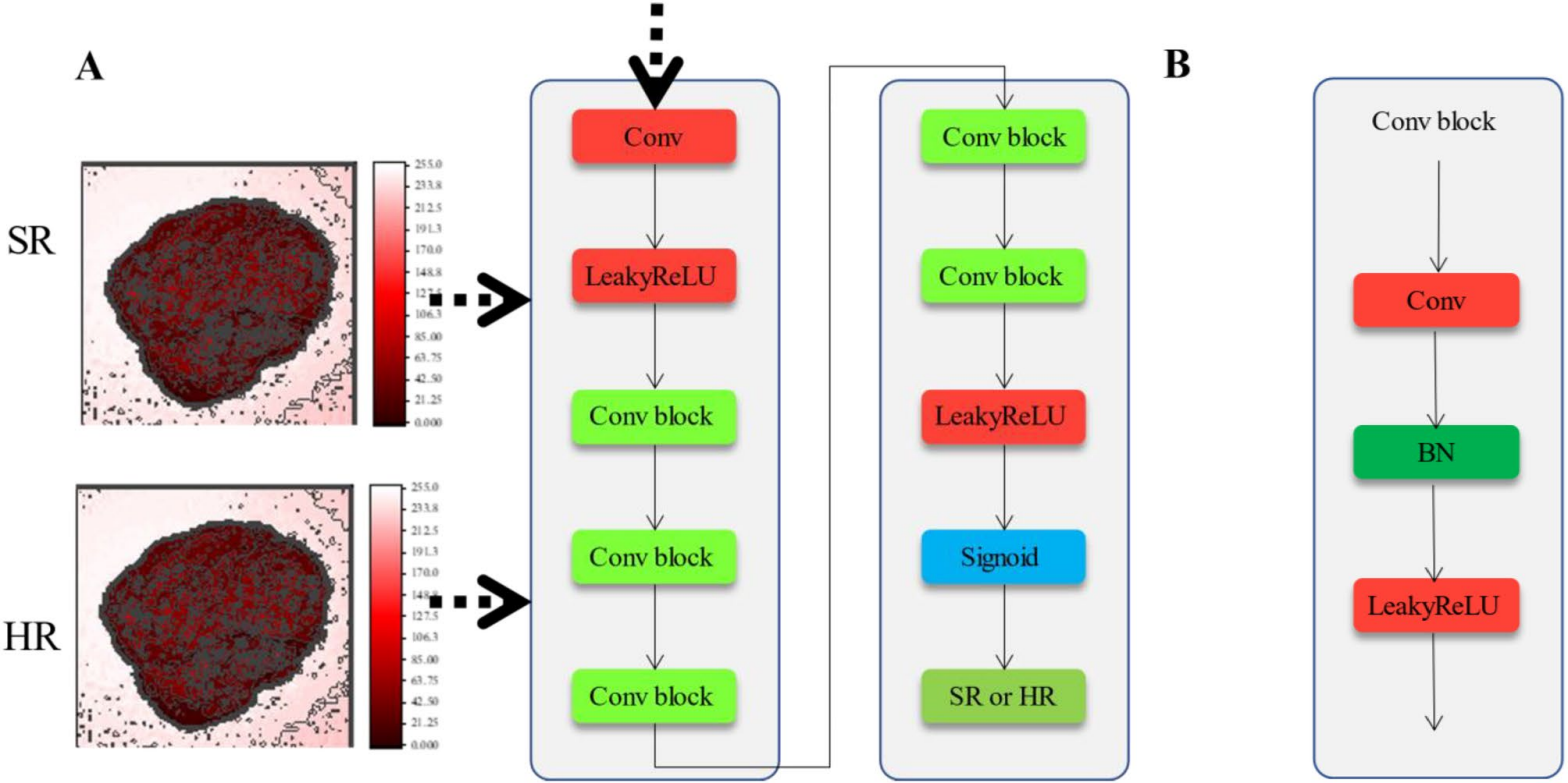
Discriminator structure. (**A**) The overall structure of the discriminator. (**B**) The structure of the Conv block.

The discriminator used in the algorithm is based on the structure proposed in Ref.^29^, as shown in Fig. 8. The relative discriminator network is essentially a binary classifier, as shown in Eq. (2).

2 $$\left. {\begin{array}{*{20}l} {D\left( {{\text{HR}}_{{\text{r}}} ,{\text{HR}}_{{\text{f}}} } \right) = \sigma \left( {I_{D} \left( {{\text{HR}}_{{\text{r}}} } \right) - E\left[ {I_{D} \left( {{\text{HR}}_{{\text{f}}} } \right)} \right]} \right)} \hfill \\ {D\left( {{\text{HR}}_{{\text{f}}} ,{\text{HR}}_{{\text{r}}} } \right) = \sigma \left( {I_{D} \left( {{\text{HR}}_{{\text{f}}} } \right) - E\left[ {I_{D} \left( {{\text{HR}}_{{\text{r}}} } \right)} \right]} \right)} \hfill \\ \end{array} } \right\},$$

where HR_r_ represents the original HR image, HR_f_ represents the SR image obtained by the reconstruction network, σ denotes the sigmoid activation function, *I*_D_ represents the parameters of the convolutional layers in the discriminator network, *E* represents the mean value of all samples in the current dataset. Assuming that the input to the discriminator is a reconstructed image HR_f_, the improved output is no longer the probability of HR_r_ being the original image, but the probability of HR_f_ being more realistic than the original image. This enables the HR image to play a more active role in training.

#### Loss function design

During the reconstruction process, there is always inherent uncertainty in the details of the image from LR to HR. In ANIMR-GAN, the loss function of the reconstruction network *G* is designed to consist of four parts: pixel-level loss, perceptual loss, adversarial loss, and cycle consistency loss, as shown in Eq. (3).

3 $$L_{{\text{ANIMR-GAN}}} = L_{{{\text{MAE}}}} + L_{p} + L_{G} + L_{c} ,$$

where the average absolute error loss (*L*_MAE_) was used to optimize the difference between the reconstructed network and the original image pixel-level loss. Perceptual loss *L*_p_ is used to optimize the perceptual difference between the reconstructed image and the original image, to make the reconstructed image look more realistic. The adversarial loss *L*_G_ is optimized by generating the faction results of discriminator in the course of the countermeasure network training. Cyclic consistency loss *L*_c_ is to ensure the authenticity of the results generated by the reconstructed network and the degraded network in the double cyclic structure.

##### Pixel-wise loss

The algorithm uses mean absolute error (MAE) loss instead of mean square error loss as the training target to obtain the ideal Peak Signal to Noise Ratio (PSNR), as shown in Eq. (4).

4 $${\text{MAE}} = \frac{1}{H \times W}\sum\limits_{i = 1}^{H} {\sum\limits_{j = 1}^{W} {\left| {F_{i,j} - Y_{i,j} } \right|} } ,$$

where the variables *H* and *W* represent the size of the feature map, *i* and *j* represent the coordinates of the pixel. *F*_*i*,*j*_ represents the pixel grayscale value of the SR image at that point, and *Y*_*i*,*j*_ represents the pixel grayscale value of the original HR image at that point. The calculation error is modified from calculating the square of the difference to calculating the absolute difference, reducing the computational complexity during iteration, improving the convergence speed of the model, and alleviating the problem of over-blurring in the SR image.

##### Perceptual loss

Perceptual loss originally from Stanford university Li Fei team^30^, specifically for SR task. The perceptual loss function uses the VGG19 network for feature extraction. Since the perception gap between the two images can be measured more comprehensively by using the inactive feature map, the feature map before the ReLU activation layer is selected to calculate the loss. The perceived loss function is shown in Eq. (5).

5 $$L_{{\text{p}}} = \frac{1}{H \times W}\sum\limits_{i = 1}^{H} {\sum\limits_{j = 1}^{W} {\left( {\varphi \left( {F_{i,j} } \right) - \varphi \left( {Y_{i,j} } \right)} \right)^{2} } } ,$$

where *H* and *W* is the size of image, subscript *i*,*j* represent the coordinates of pixel points; *φ* represent the VGG19 network; *F*_*i,j*_ is the pixel gray value of the reconstructed image at the point; *Y*_*i,j*_ is the pixel gray value of the HR original image at the point.

##### Adversarial loss

The training process of generating adversarial network is the iterative learning process of generator and discriminator in game adversarial. The adversarial loss function of relative discriminator is shown in the Eq. (6).

6 $$L_{D} = - E_{xr} \left[ {\lg \left( {D\left( {x_{r} ,x_{f} } \right)} \right)} \right] - E_{xf} \left[ {\lg \left( {1 - D\left( {x_{f} ,x_{r} } \right)} \right)} \right],$$

where *E* represents taking the average of all the images in the current batch, *D* represents the discriminator network, *x*_r_ represents the real image, and *x*_f_ represents the SR image. The adversarial loss function of the generator is shown in Eq. (7).

7 $$L_{G} = - E_{xr} \left[ {\lg \left( {1D\left( {x_{r} ,x_{f} } \right)} \right)} \right] - E_{xf} \left[ {\lg \left( {D\left( {x_{f} ,x_{r} } \right)} \right)} \right].$$

The adversarial loss of the generator not only includes the reconstructed image but also the HR original image, thus promoting the training of the reconstruction network. In SRGAN, since there is a lack of real HR images for training, only reconstructed images can be used for training. As a result, traditional GAN models are not effective because there are no real images for the discriminator to compare with. Therefore, using a relative discriminator is a more appropriate choice. The relative discriminator can not only distinguish between reconstructed images and HR original images but also help the generator to better learn the mapping relationship between them. In this way, the generator can learn how to generate more realistic images, thereby improving the learning ability of the reconstruction network.

##### Cycle consistency loss

Through adversarial loss, the reconstruction network, degradation network, and discriminator can be trained separately. However, in the process of reconstruction, the network may generate different outputs based on the fixed input of MSI, leading to a one-to-many mapping between the LR and HR images. To ensure that the input image’s feature information is not lost, cycle consistency loss is introduced in the cyclic structure. This loss can ensure that the input X remains close to the output y after passing through a cycle, as shown in Eq. (8).

8 $$y = F(G(X)) \approx X.$$

The cycle consistency loss is obtained by summing the average absolute error between the inputs and outputs of the two cycle structures, as shown in Eq. (9).

9 $${\text{L}}_{\text{cycle }}\text{(}\text{G,F}\text{) \,= \,}{\text{MAE}}\text{(}{\text{F}}\text{(}{\text{G}}\text{(}{\text{X}}\text{))}-{\text{X}}\text{)+}{\text{MAE}}\text{(}{\text{G}}\text{(}{\text{F}}\text{(}{\text{Y}}\text{))}-{\text{Y}}\text{)}\text{,}$$

where MAE represents mean absolute error, *G* represents the reconstruction network, and *F* represents the degradation network.

### Evalution index

This article uses two evaluation parameters, namely, structural similarity (SSIM) and peak signal-to-noise ratio (PSNR), to assess the super-resolution reconstruction algorithm^31^. SSIM is a subjective measure based on three relatively independent factors: luminance, contrast, and structure. It is used to quantify the structural similarity of the generated image, and its value ranges from 0 to 1. A higher value indicates a smaller error between the reconstructed image and the original image. The definition of SSIM is as follows Eq. (10).

10 $$SSIM\left( {I_{SR} ,I_{HR} } \right) = \frac{{\left( {2\mu_{{I_{SR} }} \mu_{{I_{HR} }} + c_{1} } \right)\left( {2\sigma_{{I_{SR} I_{HR} }} + c_{2} } \right)}}{{\left( {\mu_{{I_{SR} }}^{2} + \mu_{{I_{HR} }}^{2} + c_{1} } \right)\left( {\sigma_{{I_{SR} }}^{2} + \sigma_{{I_{HR} }}^{2} + c_{2} } \right)}}.$$

PSNR, a sensitive error-based image evaluation metric, is mainly calculated based on the mean square error (MSE) between the original and reconstructed images. It is commonly used to measure the similarity between the original and reconstructed images, with higher values indicating smaller errors between them. The formula for calculating PSNR is as follows in Eq. (11).

11 $$PSNR = 10\log_{10} \left( {\frac{{x_{{\max^{2} }} }}{{\frac{1}{l \times m}\sum_{i = 1}^{l} {\sum_{j = 1}^{m} {\left\| {I_{SR} (i,j) - I_{HR} (i,j)} \right\|^{2} } } }}} \right),$$

where *n* represents the number of bits per pixel, which is typically 8. *l* and *m* represent the dimensions of the image, and *I*_SR(*i*,*j*)_ and *I*_HR(*i*,*j*)_ denote the pixel values of the SR image and HR image at position (*i*,*j*). $\mu_{{I_{SR} }}$ and $\mu_{{I_{HR} }}$ are the mean values of the pixels in the SR and HR images, while $\sigma_{{I_{SR} }}$ and $\sigma_{{I_{HR} }}$ are the standard deviations of the pixels in SR and HR images. $\sigma_{{I_{SR} I_{HR} }}$ represents the covariance between the SR and HR images, *c*_1_ and *c*_2_ are constants.

### Random forest modeling

Random forest (RF) is an improved variant of the bagging integration algorithm. Based on the decision tree, the random forest model is constructed by integrating multiple decision trees. In particular, the random selection of feature attributes is introduced in the training process of the random forest. Therefore, for the same data set, the double randomness of random forest provides better generalization ability and anti-fitting ability.

After using the ANIMR-GAN method to reconstruct coal and gangue images, we plan to use the RF method to classify the reconstructed images. To achieve this, we need to determine two important parameters. Firstly, we need to set the number of decision trees in the RF, which is crucial for balancing computational resources and model performance. Secondly, we need to select the feature wavelengths of the coal and gangue MSI, identifying the key spectral lines that are relevant to coal and gangue recognition, which is essential for reducing model input and improving model robustness.

## Experimental results and analysis

This section presents a comprehensive validation of the proposed algorithm using real-world coal and gangue multispectral images (MSI). We perform a comparative analysis against various existing algorithms to assess its effectiveness. To facilitate this comparison, we down-sample the original high-resolution (HR) images by factors of 2, 4, and 8, generating corresponding low-resolution (LR) counterparts. The validation experiments are conducted on a well-equipped computational platform comprising a 64-bit Ubuntu 20.04 operating system, an Intel Core i7 processor, and an Nvidia GeForce RTX 3090 graphics card. The algorithm implementation leverages Python 3.8 and the PyTorch deep learning framework.

Experimental steps are as follows: Firstly, all the data is divided into three data sets, namely training set, test set and independent verification set. The ratio is 7:3:2. The independent validation set samples are taken from CM6 mining area. Since the selection of the training set can affect the accuracy of the model, we repeated the recognition of spectral images at different wavelengths 100 times, with each training dataset being randomly selected. Secondly, the original grayscale image is mapped to a heatmap and resized to 400 × 400 pixels. Thirdly, the image is downsampled to obtain LR images at 2×, 4×, and 8× downsampling rates, resulting in image sizes of 200 × 200, 100 × 100, and 50 × 50, respectively. During training, the L1 loss function and Adam optimizer are used, with *β*_1_ = 0.8, *β*_2_ = 0.999, and *ε* = 10^−7^. The learning rate is set to 10^−4^. At the beginning of training, the reconstruction network and degradation network are separately pre-trained for 10 rounds. Then, the trained model and discriminator are used for alternate training for 30,000 iterations. The total training time for the model is about 48 h. The specific parameters of the model are shown in Table 1.

**Table 1:** Test parameter setting.

Test parameter	Value
Image input type	RGB of heat map
size of input	200 × 200, 100 × 100, 50 × 50
Number of images per batch	32
The exponential decay rate of Adam *β*_1_	0.8
The exponential decay rate of Adam *β*_2_	0.999
Adam parameters *ε*	10^–7^
learning rate	10^–4^
Number of pre-trained rounds	10

### Determination of the number of residuals

**Figure 9:**
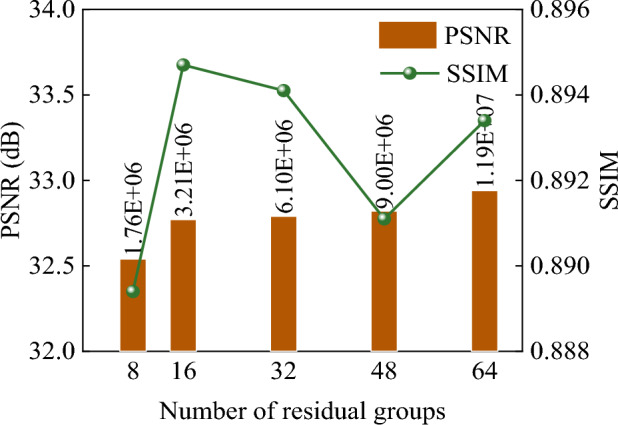
Experimental results for different numbers of RG, the optimal number of RGs for the ANIMR-GAN model was determined to be 32 based on a balance between reconstruction quality and computational efficiency. This configuration provides a good balance of performance and resource utilization, making it suitable for real-time applications where both accuracy and speed are important.

ANIMR-GAN uses a residual set to learn the mapping from LR images to HR images. The number of RG affects the overall network parameters and results. We trained different numbers of RG and tested 4 × SR on the dataset. The number of RG was set to 8, 16, 32, 48, and 64. Figure 9 shows the relationship between the number of RG and the reconstruction results. When the number of RG is 8, the SSIM and PSNR values are 0.8894 and 32.54, respectively. When the number of RG is increased from 8 to 16, the SSIM and PSNR values continue to improve, with an increase of 5.959‰ and 7.068‰ to reach 0.8947 and 32.77, respectively, indicating an improvement in the reconstruction quality. When the number of RG is increased from 16 to 48, the SSIM value shows a slight decrease, but the PSNR continues to increase, reaching 0.8911 and 32.82, respectively. When the number of RG is increased from 48 to 64, both SSIM and PSNR values increase to 0.8934 and 32.94, respectively, and the reconstruction quality reaches its best. Increasing the number of RG from 8 to 64 increases the number of parameters from 1,765,021 to 11,940,358. However, this substantial increase in the number of parameters does not significantly improve the reconstruction quality and instead wastes computational resources. Therefore, we determine the number of RG to be 32. This ensures reconstruction quality while reducing the number of parameters and improving computational speed.

### Comparison of different SR methods

In this section, we compare ANIMR with commonly used SR methods, including super resolution convolutional neural network (SRCNN), sparse coding-based network (SCN), and very deep networks for SR (VDSR). The evaluation is based on existing MSI of coal and gangue, and various metrics such as PSNR and SSIM are used to comprehensively assess the performance of each model.

The main idea of SRCNN is to divide the image into three stages: image block extraction, non-linear mapping, and image reconstruction, based on the relationship between deep learning and traditional sparse coding. These stages are then combined into a single deep convolutional neural network framework.

The VDSR is based on the SRCNN approach. However, it incorporates a VGG network structure for image classification and introduces a deeper neural network with 20 weight layers. This approach aims to establish a mapping model from low-resolution to HR images using a more profound network architecture.

The sparse coding based network (SCN) is a method that borrows from the idea of sparse representation SR. It combines the independent optimization modules of sparse representation, mapping, and sparse reconstruction into a sparse network and collaboratively optimizes these modules to obtain a globally optimal solution. SCN first obtains the sparse prior information of the image through the feature extraction layer, and then establishes a feedforward neural network SCN based on the learned iterative shrinkage and thresholding algorithm (LISTA) to realize the sparse encoding and decoding of the image. Finally, image enlargement is achieved through a cascaded network.

All of the above three methods, including the Super Resolution Convolution Neural Network (SRCNN), Very Deep Networks for super-resolution (VDSR), and SCN, are commonly used SR algorithms. The following section will compare ANIMR with these three methods, using evaluation metrics such as PSNR and SSIM to comprehensively evaluate each model.

**Figure 10:**
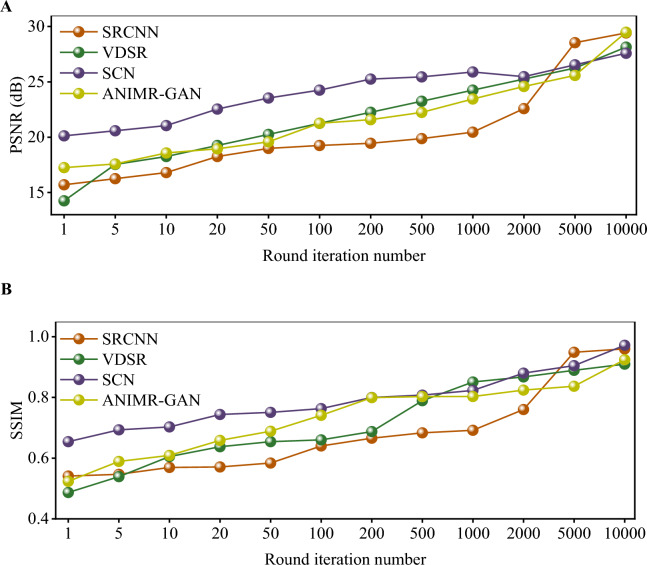
Experimental results of ANIMR-GAN and other networks with different models. (**A**) The value of PSNR. (**B**) The value of SSIM.

Different models of ANIMR, SRCNN, VDSR, and SCN were established and their reconstruction results under different iterations are shown in Fig. 10. The horizontal axis represents the iteration number of the algorithm, while the vertical axis shows the PSNR and SSIM indicators obtained from the validation dataset. The results indicate that the reconstruction results of ANIMR-GAN are significantly better than those of the simpler SRCNN and VDSR, and slightly better than those of SCN.

**Figure 11:**
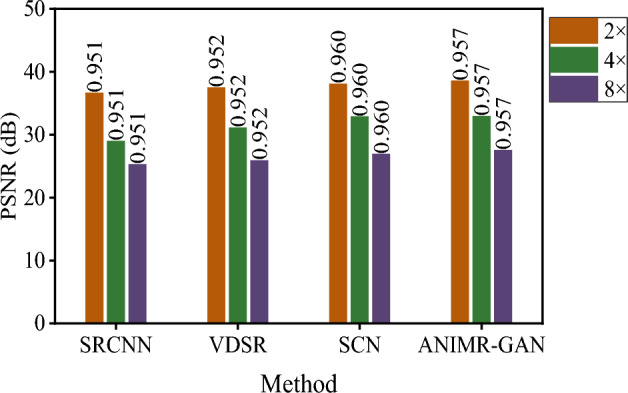
The experimental results of different SR methods. The labels on the bar chart represent the SSIM of different models.

To compare the SR performance of the four methods (SRCNN, VDSR, SCN, ANIMR-GAN) at different magnification factors, we used each method with 2×, 4×, and 8×, and calculated the SSIM and PSNR values. Figure 11 provides detailed comparison results. Overall, the SSIM and PSNR values of the reconstruction models decrease with increasing magnification factors, indicating a decrease in reconstruction performance. For the × 2 magnification model, ANIMR-GAN achieved the best PSNR of 38.61 and an SSIM of 0.957. For the × 4 magnification model, ANIMR-GAN achieved the highest SSIM and PSNR values, which were 32.97 and 0.906, respectively. SCN also performed well, with SSIM and PSNR values of 32.9 and 0.902, respectively. For the × 8 magnification model, ANIMR-GAN still performed the best, with PSNR and SSIM values of 27.56 and 0.780, respectively. To balance the computational resources and reconstruction performance, we chose a magnification factor of × 4, which ensures the reconstruction performance without a dramatic increase in the input volume of the classification model.

Based on the experimental results mentioned above, it can be concluded that the ANIMR-GAN method performs well in identifying coal and gangue images under different iteration numbers and reconstruction ratios. Compared with SRCNN, VDSR, and EDSR, it shows higher SSIM and PSNR values.

### Random forest classification method based on ANIMR-GAN

Firstly, the number of decision trees in the RF needs to be determined. Previous research has shown that the more decision trees in a RF, the better the learning performance. However, we cannot set too many decision trees, as this would be a waste of computer resources and time. We selected five typical bands, including the maximum value, minimum value, and three intermediate values at 682.92 nm, 736.25 nm, 851.48 nm, 900.95 nm, and 959.37 nm, to study the relationship between the number of decision trees in RF and accuracy.

**Figure 12:**
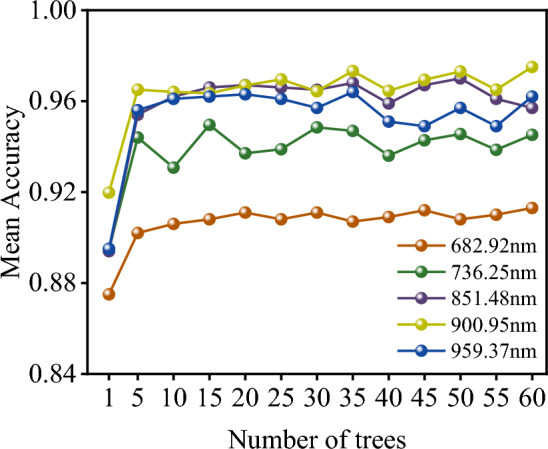
The relationship between the average recognition rate of RF model and different quantitative decision trees.

In RF, the more decision trees there are, the better the learning performance, but we cannot set too many decision trees as this would be wasteful in terms of computer resources and time. Figure 12 shows the average recognition accuracy for the selected bands at different numbers of decision trees. As the number of decision trees increases, the average accuracy also increases. However, once the number of decision trees exceeds 50, the increase in average accuracy becomes minimal, and the accuracy stabilizes. Therefore, we set the number of decision trees to 50. Additionally, we observed that the average accuracy of different spectral bands was not consistent, with 736.25 nm having a relatively large fluctuation in average recognition rate for coal and gangue, which may be related to the light source.

**Figure 13:**
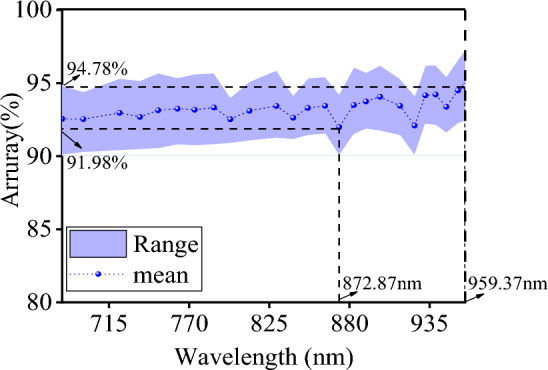
The average recognition accuracy and accuracy range corresponding to different wavelengths.

Figure 13 illustrates the range and average accuracy of identification obtained by inputting MSI of different wavelengths into the RF model. The maximum and minimum values are marked with dotted lines and labels in the figure. The trend of average accuracy is positively correlated with wavelength, i.e., higher wavelengths tend to have higher accuracy rates. The maximum average accuracy appears at 959.37 nm, while the minimum appears at 872.87 nm. This experimental result is very close to previous research^22^. Since there is a one-to-one correspondence between wavelength and functional groups of substances, 872.87 nm corresponds to the stretching and vibration of the C–O chemical bond in alcohols or phenols. We speculate that the poor accuracy at this wavelength may be due to the presence of certain alcohol-like substances in both coal and gangue.

### Comparison of classification methods for coal gangue with SR

After conducting experiments to determine the optimal number of decision trees in the RF model, it has been confirmed that the combination of ANIMR-GAN and RF can be used for identifying coal and gangue in MSI. To compare the performance of different classification methods, commonly used machine learning methods such as K-Nearest Neighbor (KNN), Support Vector Machine (SVM), Least Squares Support Vector Machines (LSSVM), and eXtreme Gradient Boosting (XGBoost) will be compared to RF. The parameter settings for each method are listed in Table S1, the parameter optimization method mainly employs grid search.

**Figure 14:**
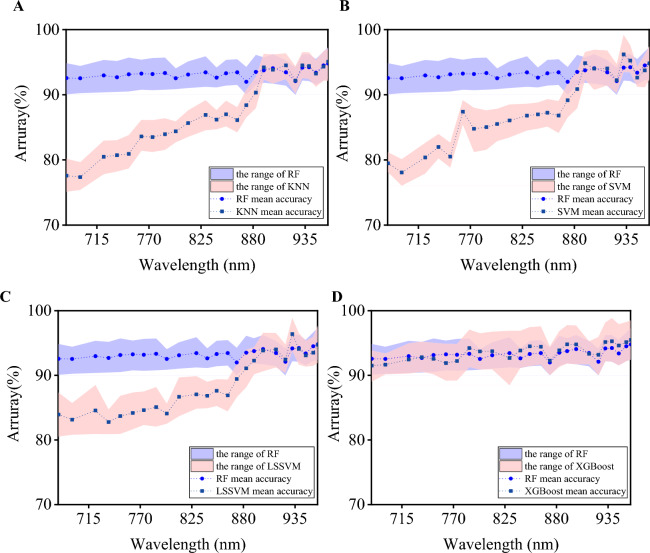
Comparison of accuracy between different methods. (**A**) KNN, (**B**) SVM, (**C**) LSSVM. (**D**) XGBoost.

Figure 14 shows the comparison results of recognition accuracy, and the following experimental conclusions are obtained.

1. To compare the classification performance of different machine learning models on the coal and gangue images reconstructed by ANIMR-GAN, KNN, SVM, LSSVM, XGBoost, and RF algorithms were used. The accuracy of each band image is different, and overall, the accuracy tends to increase with the increase in wavelength.
2. Based on the experimental results, the classification accuracy of KNN, SVM, and LSSVM was not as good as that of RF in the wavelength range of 682.92 nm to 882.95 nm. However, for wavelengths beyond 882.95 nm, the five methods showed similar classification performance. XGBoost’s classification accuracy was not significantly different from that of RF across all 25 wavelength bands, but its average accuracy was slightly lower.
3. Compared to KNN, SVM, and LSSVM, RF has a lower standard deviation, resulting in smaller fluctuations in accuracy. Although XGBoost has a similar average recognition rate to RF, its standard deviation is still larger.
4. It is worth noting that the maximum recognition rates for SVM, LSSVM, and XGBoost all correspond to the 932.08 nm wavelength, which is associated with the stretching and vibration of C–O–C chemical bonds in lipid substances. This suggests that the chemical substances at this wavelength may be of significant importance in distinguishing between coal and gangue. However, due to the precision limitations of the multispectral equipment, further research is needed to determine the specific wavelengths and corresponding chemical substances.

### Validation on independent dataset

An independent dataset consisting of 50 samples collected from the CM6 mining area was used to validate the classification performance of ANIMR-GAN combined with different modeling methods. The spectral acquisition and processing of the independent dataset followed the same procedures as the modeling dataset. Table 2 shows the classification results of the MSI dataset using five methods, including KNN, SVM, LSSVM, XGBoost, and RF. The wavelength corresponding to the highest and lowest accuracy is similar to that in Fig. 14. The average accuracy is slightly lower. RF achieves the highest average accuracy of 93.72%, which is higher than KNN, SVM, and LSSVM, and slightly higher than XGBoost’s 93.59%. This result indicates a good reconstruction and classification performance. Additionally, XGBoost’s wavelength corresponding to the lowest accuracy is also 872.87 nm, which is consistent with that of RF. The reconstruction performance varies with the magnification rate and decreases with the increase of the magnification rate.

**Table 2:** The validation results on the independent dataset.

Method	Max accuracy		Min accuracy		Mean accuracy (%)
	Wavelength (nm)	Accuracy (%)	Wavelength (nm)	Accuracy (%)	
KNN	959.37	95.78	697.42	77.35	87.52
SVM	932.08	96.19	697.42	78.07	88.19
LSSVM	946.45	96.37	736.25	82.76	88.63
XGBoost	959.37	95.46	872.87	91.49	93.59
RF	959.37	97.78	872.87	91.96	93.72

## Conclusion

The separation of coal and gangue is a crucial step in the coal mining and selection process. The use of MSI combined with ore sorting equipment for gangue separation can greatly improve mining efficiency and save on the consumption of ore sorting reagents and water resources. SR can address the problem of LR caused by equipment limitations and dust interference. In this paper, an attention mechanism is introduced to construct a multi-level residual network and a cyclic generative adversarial network structure, forming a new reconstruction method called ANIMR-GAN. This is of significant importance in accelerating the application of MSI in coal and gangue identification.

We collected 60 coal samples and 60 gangue samples from six mining areas, with samples from five of the mining areas used for model construction and research, and samples from the remaining mining area used for independent data set validation after modeling. We employed a MSI device to capture images in the 682.92–959.37 nm wavelength range. The impact of different residual combinations on the SSIM and PSNR values of the reconstruction model was compared, and 32 RB were ultimately chosen to balance computation and reconstruction efficacy. ANIMR-GAN was compared to three commonly used reconstruction algorithms, SRCNN, VDSR, and SCN, under different iteration numbers and reconstruction magnifications. The results showed that as the iteration number increased, the reconstruction performance improved. ANIMR-GAN’s reconstruction results were significantly superior to those of the simpler SRCNN and VDSR models and slightly better than SCN. In the 4× reconstruction, ANIMR-GAN had the highest SSIM and PSNR values, which were 32.97 and 0.906, respectively. To balance classification and reconstruction performance, a reconstruction magnification of 4 was chosen.

Next, we compared the multispectral RF classification models with different numbers of decision trees and different spectral bands. The results showed that the accuracy of the RF models varied depending on the spectral band used, with generally better performance observed at longer wavelengths. However, the model built using 872.87 nm had the lowest accuracy. Further analysis revealed that both coal and gangue contain certain alcohols that may have affected the modeling results. Nonetheless, further investigation is needed to confirm this finding.

Finally, we combined ANIMR-GAN with the RF algorithm and compared it with KNN, SVM, LSSVM, and XGBoost, achieving good classification performance on both the modeling and independent datasets. The highest accuracy reached 97.78%, corresponding to a wavelength of 959.37 nm, with an average accuracy of 93.72%. The results show that using the 959.37 nm band for modeling is more effective than using all 25 bands. Compared to previous studies on coal and gangue classification without SR, this approach improved accuracy by approximately 2%^11^.

Certainly, there is always room for improvement. In the next step, we will study feature extraction from the images reconstructed by ANIMR-GAN, and select combinations of bands for modeling analysis, to continuously improve the accuracy of the models we build.

### Supplementary Information

Supplementary Table S1.

## Data Availability

The data used to support the findings of this study are available from the corresponding author upon request.

## Abbreviations

SR: Image super-resolution reconstruction
MSI: Multispectral imaging
SRCNN: Super resolution convolution neural network
SCN: Sparse coding based network
VDSR: Very deep networks for super resolution
LR: Low-resolution
HR: High-resolution
RF: Random forest
GAN: Generative adversarial network
RG: Residual group
RP: Residual polymer
RB: Residual block
CAB: Channel attention block
ConCat: Convolution layers
SAB: Spatial attention block
PSNR: Peak signal to noise ratio
SSIM: Structure similarity
MAE: Mean absolute error
