# Supplementary material for: Spectral band selection and ANIMR-GAN for high-performance multispectral coal gangue classification
Source: Sci Rep. 2024 Apr 2;14:7777. doi: 10.1038/s41598-024-58379-y (PMC10987529; doi:10.1038/s41598-024-58379-y)
Supplement: Supplementary file 1 — Supplementary Table S1. [file 41598_2024_58379_MOESM1_ESM.docx]

**Suppl. Material**

**Table S1** Parameter selection after optimization about XGBoost, KNN, LSSVM

| **Method** | **Parameter** | **Implication** | **Optimal Value** | **Grid Search** |
| --- | --- | --- | --- | --- |
| KNN | *k* | The *k* points that are the least distance | 15 |  |
| LSSVM | γ | Regularized parameter | 180 | 10~300, step size is 10 |
|  | σ^2^ | RBF function parameters | 121 | 1~400, continuous |
| XGBoost | n_estimatiors | The number of child learners | 100 | [10,20,50,100,1000,2000] |
|  | learning_rate | Learning rate | 0.05 | [0.01,0.02,0.05,0.1,0.2,0.3,0.4] |
|  | max_depth | The max depth of tree | 20 | [1,3,5,7,9,12,15,20,50] |
|  | subsample | Image sampling rate | 1 | [0.1,0.2,0.3,0.4,0.5,0.6,0.7,0.8,0.9,1] |
|  | min_child_weight | Number of samples in leaf nodes | 1 | [0.1,0.3,0.5,0.7,1,3,5,7,9] |
|  | colsample_bytree | Characteristic sampling rate | 0.6 | [0.1,0.2,0.3,0.4,0.5,0.6,0.7,0.8,0.9,1] |
|  | booster | booster | ghtree | - |
|  | reg_alpha | *L*_1_ regularization coefficient | 0 | Default |
|  | reg_lambda | *L*_2_ regularization coefficient | 1 | Default |
|  | gamma | Minimum split gain | 1 | Default |
